# Predictive analysis of the relationship between nurses’ attitudes toward patient safety and missed nursing care

**DOI:** 10.1186/s12912-025-04224-0

**Published:** 2025-12-12

**Authors:** Mahadi Iddrisu, Iddrisu Mohammed Sisala, Mudasir Mohammed Ibrahim, Abubakari Wuni

**Affiliations:** 1 School of Nursing and Midwifery, Deakin University, Melbourne, Australia; 2 Anaesthesia Unit, Surgical Department, Tamale Teaching Hospital, Tamale, Ghana; 3 Department of Nursing, Nurses’ and Midwives’ Training College, Tamale, Ghana; 4 Department of Internal Medicine (M3), Tamale Teaching Hospital, Tamale, Ghana; 5 College of Nursing, University of Kentucky, Lexington, USA

**Keywords:** Attitudes, Patient safety, Missed nursing care, Nurses

## Abstract

**Background:**

Missed nursing care, defined as any aspect of required patient care that is omitted or delayed, poses significant threats to patient safety and quality outcomes. Nurses’ attitudes toward patient safety may influence their prioritization of care activities and the occurrence of missed care.

**Aim:**

This study examined the relationship between nurses’ attitudes toward patient safety and missed nursing care at Tamale Teaching Hospital, Ghana.

**Methods:**

A cross-sectional study was conducted to address the study aims. Data were collected using a structured questionnaire, comprising the Safety Attitudes Questionnaire (SAQ) and the MISSCARE survey. Spearman’s correlation and robust regression analyses were used to assess the relationship between patient safety attitudes and missed nursing care. Data analysis was performed using R version 4.2.3.

**Results:**

A total of 239 registered nurses participated. The mean attitude toward patient safety among nurses was 60.49 (SD = 16.51). Among the six dimensions, Job Satisfaction scored highest (M = 67.68, SD = 22.21), while Perception of Management scored lowest (M = 49.12, SD = 24.20). The mean level of missed nursing care was 1.74 (SD = 0.52), with participation in interdisciplinary patient care conferences being the most frequently missed activity (M = 2.09, SD = 0.84). Linear regression analysis showed that highest educational level, work experience, and unit of work were significant predictors of patient safety attitudes. Spearman’s correlation indicated significant negative relationships between missed nursing care and teamwork climate (ρ = -0.31, *p* < 0.001), safety climate (ρ = -0.30, *p* < 0.001), and job satisfaction (ρ = -0.40, *p* < 0.001). Robust regression confirmed a statistically significant negative association between overall patient safety attitude and missed nursing care (β = -0.18, SE = 0.05, t = -3.70, *p* < 0.001).

**Conclusion:**

Positive attitudes toward patient safety are associated with lower levels of missed nursing care. Interventions targeting nurses’ perceptions of teamwork, safety climate, and job satisfaction may help reduce missed care and improve patient outcomes.

## Background

Patient safety remains a top priority for healthcare institutions [1, 2] and a critical component of quality care [3]. It stands as a fundamental human requirement and is consistently emphasized in clinical practice [4]. As a key dimension of healthcare quality, patient safety is defined as “the absence of preventable harm to a patient and the reduction of risk of unnecessary harm associated with healthcare to an acceptable minimum” [5]. Nurses, who represent the largest proportion of the healthcare workforce, play a pivotal role in ensuring continuity of care, promoting health, and safeguarding patient safety [6, 7]. Their close and continuous interaction with patients positions them as frontline defenders against clinical risks, making their attitudes and practices central to preventing threats such as missed nursing care [8]. Given this crucial role, understanding how nurses’ attitudes toward patient safety influence care delivery is essential for reducing preventable harm and improving patient outcomes [3].

Patient safety outcomes reflect how individuals, teams, and organizations value and operationalize safe care, demonstrating their underlying beliefs, behaviors, and safety culture [9]. Nurses’ attitudes toward patient safety, including their perceptions of teamwork, communication, and error management, are widely recognized as determinants of safe care provision [10]. One of the most concerning manifestations of compromised patient safety is missed nursing care. Missed nursing care is defined as any aspect of required patient care that is omitted or delayed [11, 12]. Missed nursing care is associated with serious adverse events, including medication errors, infections, patient falls, prolonged hospital stays, and increased mortality [12–15].

While structural conditions such as staffing levels and workload contribute significantly to missed nursing care [16], emerging evidence shows that nurses’ safety attitudes strongly influence the extent to which essential care tasks are prioritized or left undone [10]. To explain these relationships, this study draws on the Donabedian Structure–Process–Outcome (SPO) Model, which serves as a foundational framework for evaluating healthcare quality through three components: structure, process, and outcomes [17].

The SPO Model posits that organizational structure shapes the processes of care, which in turn influence outcomes such as patient safety and completeness of care [18]. Within this model, nurses’ attitudes toward patient safety are conceptualized as a critical process factor influenced by both individual and organizational determinants, including leadership support, staffing patterns, safety culture, and team dynamics. Positive safety attitudes enhance care processes by promoting vigilance, proactive risk management, and consistent adherence to safety protocols, thereby lowering the likelihood of missed nursing care [19]. Conversely, weak or negative safety attitudes can disrupt workflow, impair communication, and diminish professional accountability, increasing the risk of delays or omissions in essential nursing activities [20, 21]. Therefore, missed nursing care emerges in the SPO framework as a process-related outcome that reflects breakdowns in care delivery and signals compromised patient safety. Supporting this, a cross-sectional study by Rahmani et al. [20] demonstrated a significant inverse relationship between nurses’ safety attitudes and the incidence of missed care, showing that nurses with more positive safety attitudes were markedly less likely to omit or postpone critical patient care tasks.

As established, missed nursing care represents a significant failure in the provision of essential healthcare services and threatens both patient safety and overall care quality [15, 22]. Frequently missed tasks often include fundamental nursing activities such as timely medication administration, regular patient repositioning, patient education, and monitoring; services that are essential for preventing complications [16, 23]. Research applying safety climate and nursing management frameworks consistently showed that environments promoting teamwork, open communication, and structured supervision are associated with lower levels of missed care, emphasizing the interplay between organizational support and individual safety attitudes [24–26].

Despite sustained global advocacy for improving patient safety [27] and international research demonstrating a clear link between nurses’ safety attitudes and missed care [20], this relationship remains insufficiently explored within Sub-Saharan Africa. Existing studies from Sub-Saharan Africa reported concerning rates of uncompleted nursing tasks, with the majority of nurses acknowledging leaving tasks undone, underscoring the universality of the issue [28–30]. In Ghana, however, most research has concentrated on patient safety more broadly, without directly examining how nurses’ safety attitudes influence missed nursing care [31–35]. This gap is particularly significant in Northern Ghana, where resource shortages, staffing limitations, and systemic organizational challenges may exacerbate patient safety risks [36].

Without empirical evidence on the relationship between nurses’ safety attitudes and missed nursing care, there is a heightened likelihood of preventable adverse patient outcomes, reduced quality of care, and compromised healthcare efficiency. This situation can adversely affect patient satisfaction, trust in healthcare services, and overall health system performance. This evidence gap prompted the researchers to design this study to examine the relationship between nurses’ attitudes toward patient safety and missed nursing care at Tamale Teaching Hospital, Ghana. Findings from this study are expected to provide critical insights into how nurses’ safety attitudes influence care delivery, inform targeted interventions to reduce missed nursing care, and guide policymakers and hospital administrators in strengthening patient safety practices within resource-limited settings in Ghana.

## Methods

### Study design

This study used a quantitative, cross-sectional design to examine the relationship between nurses’ attitudes toward patient safety and missed nursing care at Tamale Teaching Hospital, Ghana.

### Study setting

The study was conducted at Tamale Teaching Hospital (T.T.H), a major tertiary healthcare facility and the primary referral center for the five Northern regions of Ghana. The hospital has a bed capacity of 812, operates 60 clinics, and serves an average of 340 patients per day, with a total staff of 3,133 [37]. As a key provider of comprehensive, round-the-clock general and specialist medical services, TTH addresses the healthcare needs of residents in Tamale and surrounding communities. During the study period, 471 registered nurses (RNs) were working across the hospital’s critical care units, distributed as follows: Neonatal Intensive Care Unit (NICU) – 40, Intensive Care Unit (ICU) – 32, Post-Anesthesia Care Unit (PACU) – 23, Accident and Emergency (A/E) – 120, Obstetrics/Gynecology Emergency Units – 18, Pediatric Emergency Unit – 37, Maternal Intensive Care Unit (MICU) – 33, and Medical High Dependency Units (HDU) – 168.

### Study population

The study population comprised registered nurses working in the critical care units of T.T.H. Inclusion criteria were: (1) being a registered nurse, (2) having at least six months of work experience at TTH, (3) working in critical care units, and (4) providing direct patient care. Nurses on administrative duties, those on leave during the data collection period, and nursing students or interns were excluded.

### Sample size and sampling technique

The sample size for this study was calculated using Yamane’s formula for a finite population [38]:

$$\:n=\:\frac{\mathrm{N}}{1+\mathrm{N}{\left(\mathrm{e}\right)}^{2}}$$

where *n* is the required sample size, *N* is the total population of nurses (471), and *e* is the margin of error, set at 0.05 for a 95% confidence level. Using this formula, the initial sample size was 217 nurses. To account for potential non-response or attrition, a 10% buffer was added, resulting in a final target sample size of 239 nurses. To further validate this sample size, a power analysis was conducted using the “*pwr.f2.test*” function in R, following Cohen [39] framework for multiple regression. Assuming a medium effect size (f^2^ = 0.25), α = 0.05, and power = 0.80, a minimum of 33 nurses was required for a model with a single predictor, while models including eight predictors required at least 60 nurses. The final sample size of 239 thus exceeded the minimum requirement, ensuring adequate statistical power and enhancing the robustness of the study findings.

A proportionate stratified random sampling technique was used to ensure representative inclusion across all clinical departments. The nursing population in each department was enumerated, and a proportionate number of nurses were randomly selected using their ID numbers with the *sample()* function in R. This approach minimized selection bias, increased variability across clinical units, and ensured that the sample accurately reflected the overall nursing workforce.

### Data collection tool

Data were collected using a structured, pretested questionnaire comprising three sections. The first section captured demographic and professional characteristics, including age, gender, highest level of education, and work experience. Responses were recorded using multiple-choice questions.

The second section used the Safety Attitudes Questionnaire (SAQ), developed and validated by Sexton, Helmreich [40], to assess nurses’ attitudes toward patient safety. This study employed the 36-item Short-Form SAQ, based on the official scale–item structure provided by the Center for Healthcare Quality and Safety (UTHealth Houston). The SAQ includes 31 items measuring six dimensions: teamwork climate (6 items, items 1–6), safety climate (7 items, items 7–13), job satisfaction (5 items, items 15–19), stress recognition (4 items, items 20–23), perceptions of management (5 items, items 24–28), and working conditions (4 items, items 29–32). Each item was rated on a 5-point Likert scale from 1 (“Disagree strongly”) to 5 (“Agree strongly”). To calculate a 100-point scale score for a dimension (e.g., teamwork climate) for an individual participant: (1) reverse-score all negatively worded items, (2) calculate the mean of the set of items from the scale, (3) subtract 1 from the mean, and (4) multiply the result by 25. The equation is as follows: Teamwork Climate Scale Score for a Participant = (((Mean of the teamwork items) – 1) × 25). Thus, scores correspond to the original Likert scale as follows: Disagree strongly (1) = 0, Disagree slightly (2) = 25, Neutral (3) = 50, Agree slightly (4) = 75, and Agree strongly (5) = 100. The percentage of participants with a positive attitude (percent agreement) is determined as the proportion of participants scoring 75 or higher, equivalent to “Agree slightly” or higher on the original scale [41–43].

The third section used the Missed Nursing Care Questionnaire (MISSCARE Survey), developed and validated by Kalisch and Williams [44], which includes 24 items assessing the frequency of missed nursing care in areas such as patient movement, rotation, evaluation, training, discharge planning, and medication administration. Responses were recorded on a 4-point Likert scale from 1 (“I miss rarely”) to 4 (“I miss always”). Higher scores indicate a greater frequency of missed nursing care.

### Validity and reliability

Content validity of the questionnaire was established through expert review by three senior nurses (one registered anesthetist and two critical care nurses) and two patient safety researchers. The experts evaluated the questionnaire for relevance, clarity, and comprehensiveness in addressing the study objectives. Following expert validation, a pilot test was conducted with 40 nurses from Tamale Regional Hospital, who were not part of the main study sample. The pilot assessed the clarity and feasibility of the questionnaire in the study setting. No issues were identified, and the original questionnaire was retained. Internal consistency was assessed using Cronbach’s alpha based on data from the main study sample. For the Safety Attitudes Questionnaire (SAQ), the overall Cronbach’s alpha was 0.91, indicating high reliability. Reliability coefficients for the individual subscales were: teamwork climate (0.71), safety climate (0.71), job satisfaction (0.84), stress recognition (0.77), perception of management (0.85), and working conditions (0.76). The MISSCARE Survey also demonstrated excellent internal consistency, with Cronbach’s alpha of 0.94.

### Data collection procedure

Data were collected from March to May 2025, following ethical clearance and permission from hospital authorities. Prior to data collection, the research team coordinated with unit managers to schedule appropriate times to approach nurses, minimizing disruption to workflow and patient care. Nurses were primarily approached during staff meetings and shift handovers, allowing efficient access to a large number of participants without interfering with clinical responsibilities. The purpose of the study was clearly explained, and both verbal and written consent were obtained to ensure participants’ understanding and voluntary participation. Questionnaires, administered in English, were self-completed to maintain anonymity. Completed questionnaires were collected in sealed envelopes, with no identifying information recorded. To enhance response rates, gentle reminders were issued weekly over the four-week data collection period, ensuring participants did not feel pressured. The research team maintained communication with unit heads solely for logistical coordination and did not involve supervisors in questionnaire completion, minimizing potential influence on participants’ responses.

### Data analysis

**Fig. 1:**
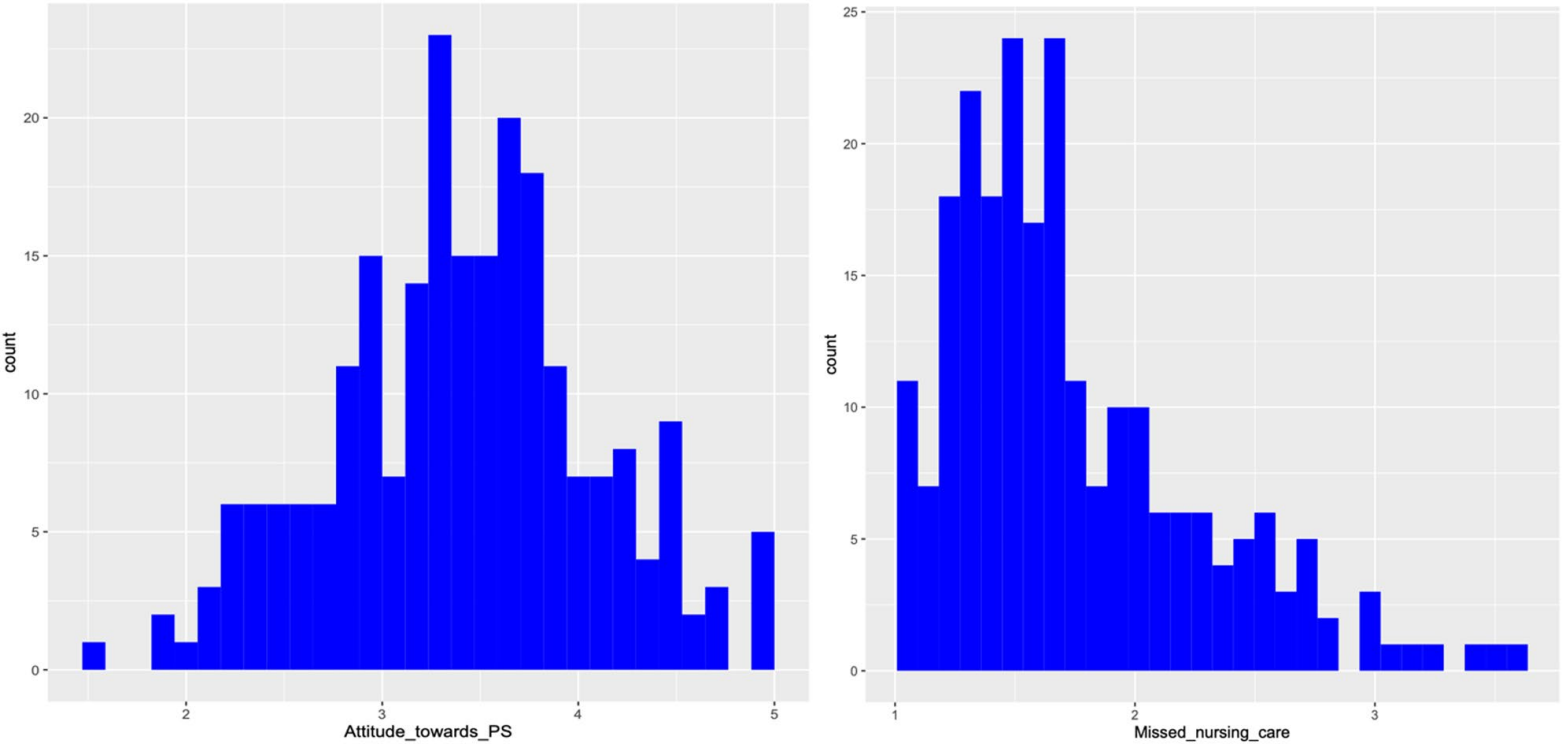
Histogram showing the distribution of nurses’ attitudes toward patient safety and missed nursing care

Data were entered and cleaned using SAS JMP Professional Statistical Software (version 17.1). Statistical analyses were conducted using R (version 4.2.3). Preliminary data screening included checks for completeness and inconsistencies. The normality of the main study variables was assessed using the Shapiro-Wilk test and visual inspection of histograms (Fig. 1). Results indicated that the attitudes toward patient safety variable was approximately normally distributed (Shapiro-Wilk test, *p* = 0.610), whereas the missed nursing care variable was not (Shapiro-Wilk test, *p* < 0.001). Prior to inferential analyses, all assumptions for linear regression, including linearity, independence of residuals, homoscedasticity, and multicollinearity, were assessed and met. A linear regression analysis was conducted to identify factors influencing nurses’ attitudes toward patient safety. Given the non-normal distribution of missed nursing care, Spearman’s rank-order correlation was used to examine the relationship between missed nursing care and the various dimensions of patient safety attitudes. To further explore the relationship between attitudes toward patient safety (predictor) and missed nursing care (outcome), a robust regression analysis was performed using the “*robustbase*” package in R. This analysis accounted for potential sociodemographic confounders by adjusting for key variables such as age, gender, marital status, highest level of education, work experience, unit of practice, work schedule, and number of patients assigned per shift. Robust regression provides reliable estimates in the presence of violations of classical linear regression assumptions, such as non-normality, heteroscedasticity, or influential outliers [45]. This approach ensured that the results were not unduly affected by outliers or the non-normal distribution of missed nursing care. All analyses were two-tailed, and statistical significance was set at *p* < 0.05.

### Ethical considerations

Ethical approval for the study was obtained from the Kwame Nkrumah University of Science and Technology (KNUST) Committee on Human Research, Publication and Ethics (Reference: CHRPE/AP/163/25). Written and verbal informed consent was obtained from each participant after explaining the study’s purpose, procedures, potential risks, and benefits. Participation was voluntary, and nurses were assured of confidentiality and anonymity. All data were stored securely and were accessible only to the research team. The study was conducted in accordance with the ethical principles outlined in the Declaration of Helsinki.

## Results

### Sociodemographic characteristics

A total of 239 nurses participated in the study, yielding a 100% response rate. The mean age of participants was 31.0 years (SD = 5.11), with nearly half (45.2%) over 30 years of age. The majority were female (52.3%) and married (58.2%). Regarding educational background, most held a bachelor’s degree (52.7%), followed by diploma holders (40.2%). Work experience was predominantly 1–5 years (51.9%). In terms of clinical practice, the largest proportion of participants worked in Medical High Dependency Units (36.4%), followed by Accident and Emergency (27.2%). Most participants (76.6%) worked rotational shifts, and the majority (70.3%) reported being assigned more than five patients per shift (Table 1).

**Table 1:** Sociodemographic characteristics of participants (*N* = 239)

Variable	Frequency	Percentage
**Age in years**	31.0 (SD: 5.11)	
**Age group**		
20–25 years	26	10.9
26–30 years	105	43.9
> 30 years	108	45.2
**Gender**		
Male	114	47.7
Female	125	52.3
**Marital status**		
Divorced	5	2.1
Married	139	58.2
Single	94	39.3
Widowed	1	0.4
**Highest level of education**		
Certificate	9	3.8
Diploma	96	40.2
Bachelor’s degree	126	52.7
Master’s degree	8	3.3
**Work experience**		
< 1 years	50	19.5
1–5 years	124	51.9
6–10 years	51	21.3
> 10 years	14	5.9
**Unit of practice**		
Accident and Emergency Unit (A/E)	65	27.2
Intensive Care Unit (ICU)	15	6.3
Neonatal Intensive Care Unit (NICU)	19	7.9
Obstetrics/Gynecology Emergency Units	10	4.2
Post-Anesthesia Care Unit (PACU)	11	4.6
Pediatric Emergency Unit	17	7.1
Maternal Intensive Care Unit (MICU)	15	6.3
Medical High Dependency Units (HDU)	87	36.4
**Work schedule**		
Day shift only	56	23.4
Rotation shifts	183	76.6
**Number of patients assigned per shift**		
1–5	71	29.7
> 5	168	70.3

**Table 2:** Summary statistics for SAQ (Safety attitudes Questionnaire) dimensions and MISSCARE survey (*N* = 239)

Variable	Min	Max	Mean	SD
Attitudes towards patient safety: *	13.99	99.29	60.49	16.51
Teamwork Climate*	8.33	100	66.82	18.07
Safety Climate*	17.86	100	64.41	15.95
Job Satisfaction*	10	100	67.68	22.21
Stress Recognition*	0	100	65.56	21.57
Perception of Management*	0	100	49.12	24.20
Working Conditions*	0	100	54.42	23.28
Missed Nursing Care	1.04	3.58	1.74	0.52

Note: *SAQ scores for each dimension are reported on a 0–100 point scale

### Patient safety attitudes

The mean attitudes toward patient safety among participants was 60.49 (SD = 16.51), with scores ranging from 13.99 to 99.29 (Table 2). Among the six dimensions assessed, Job Satisfaction had the highest mean score (M = 67.68, SD = 22.21). Within this dimension, the item “Working here is like being part of a large family” scored highest (M = 3.94, SD = 1.02), while “Morale in this clinical area is high” scored lowest (M = 3.33, SD = 1.27). Teamwork Climate followed, with a mean of 66.82 (SD = 18.07). The highest-scoring item was “It is easy for personnel here to ask questions when there is something that they do not understand” (M = 4.05, SD = 1.14), and the lowest was “In this clinical area, it is difficult to speak up if I perceive a problem with patient care” (M = 2.50, SD = 1.28). In contrast, the Perception of Management dimension received the lowest mean score among all domains (M = 49.12, SD = 24.20). The highest-rated item was “Hospital management doesn’t knowingly compromise patient safety” (M = 3.22, SD = 1.20), while “Hospital management supports my daily efforts” scored lowest (M = 2.64, SD = 1.31) (Table 3).

**Table 3:** Attitudes toward patient safety among participants (*N* = 239)

Variable	Disagree Strongly		Disagree Slightly		Neutral		Agree Slightly		Agree Strongly		Mean (SD)
	*N*	%	*N*	%	*N*	%	*N*	%	*N*	%	
**Teamwork Climate**											
1. Nurse input is well received in this clinical area.	12	5.0	29	12.1	46	19.2	87	36.4	65	27.2	3.68 (1.14)
2. In this clinical area, it is difficult to speak up if I perceive a problem with patient care.	67	28.0	68	28.5	38	15.9	49	20.5	17	7.1	2.50 (1.28)
3. Disagreements in this clinical area are resolved appropriately (i.e., not who is right, but what is best for the patient).	8	3.3	26	10.9	57	23.8	59	24.7	89	37.2	3.81 (1.14)
4. I have the support I need from other personnel to care for patients.	4	1.7	15	6.3	48	20.1	95	39.7	77	32.2	3.94 (0.96)
5. It is easy for personnel here to ask questions when there is something that they do not understand.	14	5.9	11	4.6	34	14.2	70	29.3	110	46.0	4.05 (1.14)
6. The physicians and nurses here work together as a well-coordinated team.	6	2.5	22	9.2	33	13.8	74	31.0	104	43.5	4.03 (1.08)
**Safe Climate**											
7. I would feel safe being treated here as a patient.	15	6.3	22	9.2	45	18.8	88	36.8	69	28.9	3.73 (1.15)
8. Medical errors are handled appropriately in this clinical area.	7	2.9	29	12.1	60	25.1	83	34.7	60	25.1	3.66 (1.07)
9. I know the proper channels to direct questions regarding patient safety in this clinical area.	2	0.8	14	5.9	49	20.5	99	41.4	75	31.4	3.96 (0.91)
10. I receive appropriate feedback about my performance.	12	5.0	33	13.8	76	31.8	85	35.6	33	13.8	3.39 (1.04)
11. In this clinical area, it is difficult to discuss errors.	40	16.7	62	25.9	61	25.5	60	25.1	16	6.7	2.78 (1.17)
12. I am encouraged by my colleagues to report any patient safety concerns I may have.	9	3.8	16	6.7	52	21.8	101	42.3	61	25.5	3.79 (1.01)
13. The culture in this clinical area makes it easy to learn from the errors of others.	9	3.8	22	9.2	64	26.8	82	34.3	62	25.9	3.69 (1.07)
**Job Satisfaction**											
14. I like my job.	14	5.9	14	5.9	62	25.9	65	27.2	84	35.1	3.80 (1.16)
15. Working here is like being part of a large family.	1	0.4	26	10.9	47	19.7	76	31.8	89	37.2	3.94 (1.02)
16. This is a good place to work.	13	5.4	19	7.9	66	27.6	78	32.6	63	26.4	3.66 (1.11)
17. I am proud to work in this clinical area.	11	4.6	13	5.4	61	25.5	84	35.1	70	29.3	3.79 (1.06)
18. Morale in this clinical area is high.	27	11.3	28	11.7	79	33.1	48	20.1	57	23.8	3.33 (1.27)
**Stress Recognition**											
19. When my workload becomes excessive, my performance is impaired.	5	2.1	29	12.1	43	18.0	94	39.3	68	28.5	3.80 (1.04)
20. I am less effective at work when fatigued.	13	5.4	19	7.9	47	19.7	90	37.7	70	29.3	3.77 (1.12)
21. I am more likely to make errors in tense or hostile situations.	12	5.0	46	19.2	60	25.1	66	27.6	55	23.0	3.44 (1.18)
22. Fatigue impairs my performance during emergency situations (e.g., emergency resuscitation, seizure).	18	7.5	32	13.4	56	23.4	85	35.6	48	20.1	3.47 (1.17)
**Perception of Management**											
23. Hospital management supports my daily efforts.	63	26.4	50	20.9	57	23.8	46	19.2	23	9.6	2.64 (1.31)
24. Hospital management doesn’t knowingly compromise patient safety.	28	11.7	27	11.3	89	37.2	54	22.6	41	17.2	3.22 (1.20)
25. Hospital management is doing a good job.	42	17.6	38	15.9	69	28.9	67	28.0	23	9.6	2.96 (1.23)
26. Problem personnel are dealt with constructively by our hospital management.	28	11.7	38	15.9	79	33.1	68	28.5	26	10.9	3.11 (1.15)
27. I get adequate, timely info about events that might affect my work, from hospital management.	38	15.9	54	22.6	74	31.0	44	18.4	29	12.1	2.88 (1.23)
**Working Conditions**											
28. The levels of staffing in this clinical area are sufficient to handle the number of patients.	61	25.5	44	18.4	46	19.2	56	23.4	32	13.4	2.81 (1.39)
29. This hospital does a good job of training new personnel.	16	6.7	49	20.5	66	27.6	63	26.4	45	18.8	3.30 (1.18)
30. All the necessary information for diagnostic and therapeutic decisions is routinely available to me.	28	11.7	60	25.1	67	28.0	55	23.0	29	12.1	2.99 (1.20)
31. Trainees in my discipline are adequately supervised.	10	4.2	26	10.9	64	26.8	86	36.0	53	22.2	3.61 (1.07)

### Missed nursing care

The mean missed nursing care score was 1.74 (SD = 0.52), with scores ranging from 1.04 to 3.58 (Table 2). Among the 24 care items assessed, the highest mean score was observed for participation in interdisciplinary patient care conferences (M = 2.09, SD = 0.84). In contrast, clinical and safety-related tasks were less frequently missed, with vital signs assessment as ordered recording the lowest mean score (M = 1.48, SD = 0.73) (Table 4).

**Table 4:** Missed nursing care among participants (*N* = 239)

Variable	I miss rarely		I miss occasionally		I miss usually		I miss always		Mean (SD)
	*N*	%	*N*	%	*N*	%	*N*	%	
1. Participating in interdisciplinary patient care conferences	59	24.7	117	49.0	46	19.2	17	7.1	2.09 (0.84)
2. Setting up meals for a patient who feed themselves	85	35.6	93	38.9	42	17.6	19	7.9	1.98 (0.92)
3. Assist with toileting needs within 5 min of request	96	40.2	92	38.5	36	15.1	15	6.3	1.87 (0.88)
4. Feeding patient when the food is still warm	95	39.7	93	38.9	39	16.3	12	5.0	1.86 (0.86)
5. Mouth care	89	37.2	98	41.0	35	14.6	17	7.1	1.92 (0.89)
6. Patient bathing/skincare	91	38.1	94	39.3	40	16.7	14	5.9	1.90 (0.88)
7. Turning patient every 2 h	104	43.5	93	38.9	31	13.0	11	4.6	1.78 (0.84)
8. Emotional support to patient and/or family	104	43.5	96	40.2	25	10.5	14	5.9	1.78 (0.85)
9. Assess the effectiveness of medications	100	41.8	105	43.9	24	10.0	10	4.2	1.76 (0.79)
10. Patient teaching about procedures, tests, and other diagnostic studies	96	40.2	114	47.7	21	8.8	8	3.3	1.75 (0.75)
11. Focused reassessments according to patient condition	107	44.8	96	40.2	28	11.7	8	3.3	1.74 (0.79)
12. Washing hands before caring	147	61.5	65	27.2	16	6.7	11	4.6	1.54 (0.81)
13. Ambulation 3 times per day or as ordered	71	29.7	107	44.8	53	22.2	8	3.3	1.99 (0.80)
14. Skin ulcer caring	129	54.0	86	36.0	19	7.9	5	2.1	1.58 (0.72)
15. Teach patients about plans for their care after discharge and when to call after discharge	135	56.5	72	30.1	26	10.9	6	2.5	1.59 (0.78)
16. Response to call light is initiated within 5 min	105	43.9	106	44.4	24	10.0	4	1.7	1.69 (0.71)
17. PRN medication requests acted on within 15 min	129	54.0	74	31.0	33	13.8	3	1.3	1.62 (0.76)
18. General evaluation of the patient in each shift	116	48.5	96	40.2	22	9.2	5	2.1	1.64 (0.73)
19. Intravenous/ central line site care and assessments according to hospital policy	117	49.0	78	32.6	36	15.1	8	3.3	1.73 (0.84)
20. Medications administered within 30 min before or after the scheduled time	135	56.5	73	30.5	22	9.2	9	3.8	1.60 (0.80)
21. Monitoring intake/output	125	52.3	85	35.6	24	10.0	5	2.1	1.61 (0.75)
22. Vital signs assessed as ordered	154	64.4	61	25.5	19	7.9	5	2.1	1.48 (0.73)
23. Full documentation of all necessary data	147	61.5	66	27.6	20	8.4	6	2.5	1.52 (0.75)
24. Blood glucose control with a glucometer	142	59.4	67	28.0	21	8.8	9	3.8	1.57 (0.80)

### Factors affecting patient safety attitudes

Bivariate linear regression analysis showed significant associations between patient safety attitudes and variables such as highest level of education, work experience, and unit of practice. Nurses holding a bachelor’s degree had significantly lower patient safety attitudes compared to those holding certificates (Crude β = -0.50, 95% CI: -0.94 to -0.05, *p* = 0.029). Similarly, nurses with 6–10 years of work experience reported lower safety attitudes than those with more than 10 years of experience (Crude β = -0.43, 95% CI: -0.82 to -0.03, *p* = 0.031). Nurses working in the Accident & Emergency (A&E) Unit also had significantly lower safety attitudes compared to those in the Medical High Dependency Unit (HDU) (Crude β = -0.48, 95% CI: -0.71 to -0.24, *p* < 0.001).

In the multivariate model, after adjusting for potential confounders, education level and unit of practice remained significantly associated with patient safety attitudes. Compared to certificate holders, nurses with a diploma (Adjusted β = -0.50, 95% CI: -0.96 to -0.03, *p* = 0.038), bachelor’s degree (Adjusted β = -0.62, 95% CI: -1.07 to -0.17, *p* = 0.001), and master’s degree (Adjusted β = -0.69, 95% CI: -1.34 to -0.02, *p* = 0.042) had significantly lower patient safety attitudes. Additionally, nurses in the A&E Unit continued to exhibit lower safety attitudes compared to those in the Medical HDU (Adjusted β = -0.47, 95% CI: -0.71 to -0.22, *p* < 0.001) (Table 5).

**Table 5:** Simple and multivariable linear regression analysis of variables affecting patient safety attitudes among participants (*N* = 239)

Variable	Crude β (95% CI)	*P*-value	Adjusted β (95% CI)	*P*-value
**Age in years**	0.01 (-0.01–0.03)	0.326	0.01 (-0.01-0.03)	0.431
**Gender**				
Female	1			
Male	-0.08 (-0.24–0.09)	0.373	0.04 (-0.14-0.21)	0.677
**Marital status**				
Widowed	1			
Married	-0.32 (-1.62–0.98)	0.627	0.25 (-1.11–1.61)	0.721
Single	-1.43 (-3.26–0.41)	0.127	0.44 (-0.94–1.82)	0.528
Divorced	-0.04 (-1.46–1.38)	0.953	0.51 (-0.93–1.96)	0.488
**Highest level of education**				
Certificate	1			
Diploma	-0.34 (-0.78–0.11)	0.142	-0.50 (-0.96 – -0.03)	0.038*
Bachelor’s degree	-0.50 (-0.94 – -0.05)	0.029*	-0.62 (-1.07 – -0.17)	0.001*
Master’s degree	-0.30 (-0.95–0.35)	0.363	-0.69 (-1.34 – -0.02)	0.042*
**Work experience**				
> 10 years	1			
6–10 years	-0.43 (-0.82 – -0.03)	0.031*	-0.26 (-0.69–0.17)	0.233
1–5 years	-0.31 (-0.68–0.05)	0.091	-0.22 (-0.67–0.22)	0.313
< 1 years	-0.21 (-0.61–0.18)	0.283	-0.12 (-0.59–0.36)	0.621
**Unit of practice**				
Medical High Dependency Units (HDU)	1			
Accident & Emergency Unit (A/E)	-0.48 (-0.71 – -0.24)	< 0.001*	-0.47 (-0.71 – -0.22)	< 0.001*
Intensive Care Unit (ICU)	-0.12 (-0.43–0.20)	0.466	-0.12 (-0.44–0.20)	0.468
Neonatal Intensive Care Unit (NICU)	0.06 (-0.23–0.35)	0.674	0.05 (-0.26–0.36)	0.754
Obstetrics/Gynecology Emergency Units	0.02 (-0.34–0.36)	0.945	-0.01 (-0.37–0.35)	0.940
Post-Anesthesia Care Unit (PACU)	-0.17 (-0.56–0.21)	0.367	-0.19 (-0.59–0.21)	0.344
Pediatric Emergency Unit	-0.29 (-0.61–0.03)	0.076	-0.27 (-0.60–0.06)	0.110
Maternal Intensive Care Unit (MICU)	0.12 (-0.26–0.51)	0.532	0.10 (-0.30–0.49)	0.621
**Work schedule**				
Rotation shifts	1			
Day shift only	0.07 (-0.12–0.28)	0.470	0.05 (-0.17–0.27)	0.668
**Number of patients assigned per shift**				
> 5	1			
1–5	0.09 (-0.10–0.27)	0.334	0.03 (-0.16–0.24)	0.741

Note: *Statistically significant at *p* < 0.05

### Relationship between dimensions of patient safety attitude and missed nursing care

Spearman’s correlation analysis was conducted to examine the associations between the dimensions of patient safety attitudes and missed nursing care. Significant negative correlations were observed between missed nursing care and teamwork climate (ρ = -0.31, *p* < 0.001), safety climate (ρ = -0.30, *p* < 0.001), and job satisfaction (ρ = -0.40, *p* < 0.001), indicating that higher scores in these dimensions were associated with lower levels of missed nursing care (Table 6).

**Table 6:** Spearman correlation analysis of the association between patient safety attitude dimensions and missed nursing care among participants (*N* = 239)

Variable	Missed Nursing Care	
	Coefficient (ρ)	*P*-value
Teamwork Climate	-0.31	0.001*
Safety Climate	-0.30	0.001*
Job Satisfaction	-0.40	0.001*
Stress Recognition	-0.02	0.934
Perception of Management	-0.10	0.488
Working Conditions	-0.05	0.934
Note: *Statistically significant at *p* < 0.05		

### Relationship between attitudes toward patient safety and missed nursing care

A robust regression analysis was conducted to examine the predictive relationship between nurses’ attitudes toward patient safety and missed nursing care, controlling for sociodemographic factors such as age, gender, marital status, highest level of education, work experience, unit of practice, work schedule, and number of patients assigned per shift. The regression model was statistically significant. The intercept (β₀) was 2.13 (SE = 0.17, t = 12.08, *p* < 0.001). Attitudes toward patient safety significantly and negatively predicted missed nursing care (β = -0.18, SE = 0.05, t = -3.70, *p* < 0.001), indicating that for each one-unit increase in positive patient safety attitude, the predicted level of missed nursing care decreases by 0.18 units (Table 7).

**Table 7:** Robust regression analysis of the relationship between patient safety attitudes and missed nursing care among participants (*N* = 239)

Predictor Variable (s)	β	SE	t	*P*-value
Intercept (β₀)	2.13	0.17	12.08	< 0.001
Attitudes toward patient safety	-0.18	0.05	-3.70	< 0.001*

Note: β = unstandardized regression coefficient; SE = standard error; t = t-statistic. *p* < 0.05 indicates statistical significance. The regression model controlled for all sociodemographic variables

## Discussion

Missed nursing care poses a significant threat to patient care quality. Creating a work environment that fosters strong patient safety is essential, as leadership styles and workplace roles substantially influence the occurrence of missed nursing care [46]. This study, guided by the Donabedian Structure–Process–Outcome (SPO) Model, investigated the relationship between nurses’ attitudes toward patient safety and missed nursing care at Tamale Teaching Hospital, Ghana.

The findings showed that the mean Safety Attitudes Questionnaire (SAQ) score among nurses was 60.49 (SD = 16.51). Participants scored highest on the job satisfaction dimension, suggesting that nurses generally feel fulfilled and positive about their roles. In contrast, Rahmani et al. [20] reported a lower overall SAQ score of 53.19 (SD = 18.71), with stress recognition being the highest-rated dimension. This indicates that participants in their study were more aware of the impact of fatigue, workload, and personal stress on performance. The divergence in highest-rated dimensions between the two studies may reflect contextual differences in work environments, leadership styles, and safety culture priorities across healthcare systems.

The study findings indicated that participants with more than 10 years of work experience reported significantly more positive attitudes toward patient safety compared to those with less than 10 years of experience. This suggests that prolonged exposure to clinical practice may strengthen nurses’ understanding of safety standards, reinforce adherence to protocols, and enhance their confidence in identifying and preventing errors. Consistent with this, Şahin, Ayhan [47], Biresaw, Asfaw [48] and Suganda, Hariyati [49] also found that participants with over 10 years of experience demonstrated higher patient safety attitudes. These studies suggest that older, more experienced nurses possess greater knowledge and expertise, which contributes to stronger safety culture practices. Additionally, more experienced staff are generally expected to maintain professionalism regarding patient safety [50], as extended experience and training enhance the likelihood of staying up to date, maintaining consistent presence at work, and adhering to safety protocols. However, Abu-El-Noor, Hamdan [51] reported a different finding, observing no significant association between work experience and attitudes toward patient safety among the study population. This difference may be due to variations in study settings. Nurses working in institutions with less structured safety programs may not develop the same positive attitudes regardless of experience.

The study further revealed that participants with a bachelor’s degree exhibited significantly lower patient safety attitudes compared to those holding certificates. In contrast, Brasaite, Kaunonen [52] reported that degree holders were more likely to have positive patient safety attitudes than those with lower qualifications, such as a diploma or below. Similarly, Tirgar, Hosseinabadi [53] found that participants with higher education levels demonstrated more positive safety attitudes. This discrepancy may be explained by the fact that in some resource-constrained settings, nurses with higher qualifications are often assigned more administrative responsibilities, heavier workloads, or leadership tasks, which can increase stress and reduce their perceived ability to ensure safety. Higher-educated nurses may also be more aware of systemic safety gaps, making them more critical of existing practices and consequently reporting lower attitudes.

Among the individual safety attitude dimensions, job satisfaction received the highest rating. This finding aligns with previous study by Rahmani et al. [24] who reported that stress recognition was the item with the highest rating. Nurses with higher educational qualifications also tend to express greater satisfaction with being part of a larger workforce and enjoy their roles more. Brasaite, Kaunonen [52] further noted that healthcare professionals who receive ongoing education and role-related information are more likely to experience higher job satisfaction. Importantly, satisfaction with working conditions and overall job satisfaction significantly influence missed nursing care, affecting both its occurrence and severity [54]. Moreover, Dirgar, Berşe [50] reported that reduced job satisfaction decreases nurses’ productivity, thereby lowering quality of care and placing patients at risk.

Teamwork climate received the second-highest perception rating, consistent with findings from several studies [55–57]. A supportive environment in which staff can freely ask questions and colleagues provide encouragement helps foster individual performance, reduce job pressure, and enhance motivation [58, 59]. On the contrary, Al-Mugheed, Bayraktar [7] reported that teamwork ranked third among participants in Northern Cyprus. Lima, Silva [57] further confirmed that multidisciplinary discussions, mutual support, and task delegation reduce the occurrence of missed nursing care by promoting cooperation, improving information exchange, and encouraging shared reflections on therapeutic approaches across different professional groups involved in patient care.

According to the study’s results, the perception of management dimension received the lowest mean score among participants. This might be due to inadequate daily support, untimely communication, and poor managerial performance. Similar findings have been documented in studies showing that negative perceptions of management are associated with higher levels of missed nursing care [46, 60, 61]. Effective managers cultivate supportive environments that minimize intimidation and scapegoating while fostering open communication. Research by Kim, Yoo [46] and Lake, French [62] demonstrated that positive work environments significantly reduce missed nursing care when compared to hostile or stressful conditions. However, Bottcher, Abu-El-Noor [63] reported higher positive ratings of management perceptions among nurses, indicating that managerial effectiveness may vary across healthcare settings.

Overall, the levels of missed nursing care were low, with participation in interdisciplinary patient care conferences identified as the most frequently missed activity. In contrast, hand hygiene, medication administration, vital signs assessment, and blood glucose monitoring were rarely missed, reinforcing the consistency of direct patient assessment and contact. These findings differ from those of other studies that reported more frequent lapses in these areas [60, 64]. One possible explanation for this discrepancy is that differences in staffing levels or workload may influence the extent to which basic nursing tasks are consistently performed.

The analysis also showed a negative correlation between missed nursing care and several patient safety attitude dimensions, consistent with findings from Kim, Yoo [46] and Moustafa and Abd-Elmoghith [65]. For instance, teamwork, job satisfaction, and safety climate demonstrated inverse relationships with missed care. Plevová, Zeleníková [54] similarly reported that nurses with higher job satisfaction recorded fewer instances of missed nursing care. Zeleníková, Gurková [59] also observed that lower job satisfaction and weaker teamwork were associated with higher levels of missed care. Conversely, Bragadóttir, Kalisch [66] found a significant positive correlation between teamwork and missed nursing care. Despite these mixed findings, the regression analysis in the present study confirmed a statistically significant negative association between patient safety attitudes and missed nursing care, indicating that nurses with positive safety attitudes were less likely to omit essential care activities. This is in agreement with past study by Rahmani et al. [24].

### Limitations

This study has several limitations. Firstly, the cross-sectional design prevents causal inferences between patient safety attitudes and missed nursing care. Second, the use of self-reported instruments may have introduced response, recall, and social desirability biases. Third, the study was conducted in a single tertiary healthcare facility, which may limit the generalizability of the findings to other hospitals or regions. Moreover, unmeasured contextual factors such as workload intensity, staffing ratios, and leadership styles could have influenced both safety attitudes and missed care but were not assessed. Lastly, potential variations in how participants interpreted questionnaire items may have contributed to measurement inconsistencies.

## Conclusion

This is the first study of its kind conducted in Ghana to examine the relationship between nurses’ attitudes toward patient safety and missed nursing care. The study provides important evidence demonstrating a significant negative relationship between nurses’ attitudes toward patient safety and the occurrence of missed nursing care. Nurses who reported more positive safety attitudes, particularly in domains such as teamwork climate, safety climate, and job satisfaction, were less likely to leave essential nursing tasks unfinished. In addition, factors such as the highest level of education, years of work experience, and unit of assignment were found to significantly influence safety attitudes. These findings collectively highlight the critical role of promoting a strong and supportive patient safety culture as a strategic pathway to minimizing missed care and improving the overall quality of nursing practice and patient outcomes.

### Practical implications

The findings of this study suggest several strategies to enhance patient safety in clinical settings. Nurses with higher educational qualifications exhibited lower patient safety attitudes than certificate-holding nurses, highlighting the need for targeted interventions such as tailored training, continuous professional development, and mentorship programs to align attitudes across all educational levels. Nurses in the accident and emergency unit demonstrated particularly low safety attitudes, underscoring the need for context-specific strategies, including stress management initiatives, improved teamwork, and structured safety protocols suited for high-pressure environments. Additionally, the negative associations observed between teamwork climate, safety climate, job satisfaction, and missed nursing care indicate that strengthening team communication, supportive management practices, and employee satisfaction can help reduce missed care. Overall, implementing interventions that address both individual- and unit-level factors is essential to improving adherence to patient safety practices and enhancing patient outcomes.

## Data Availability

Data supporting the findings of this study are available from the corresponding author upon reasonable request.
